# Reduction of depression-like behavior in rat model induced by ShRNA targeting norepinephrine transporter in locus coeruleus

**DOI:** 10.1038/s41398-020-0808-8

**Published:** 2020-05-04

**Authors:** Xiangdong Du, Ming Yin, Lian Yuan, Guangya Zhang, Yan Fan, Zhe Li, Nian Yuan, Xiaoli Lv, Xueli Zhao, Siyun Zou, Wei Deng, Thomas R. Kosten, Xiang Yang Zhang

**Affiliations:** 1 Suzhou Psychiatric Hospital, The Affiliated Guangji Hospital of Soochow University, Suzhou, China; 2 Department of Psychiatry and Psychiatric Laboratory, State Key Laboratory of Biotherapy, West China Hospital, Sichuan University, Chengdu, China; 3 Department of Psychiatry and Behavioral Sciences, Baylor College of Medicine, Houston, TX USA; 4 CAS Key Laboratory of Mental Health, Institute of Psychology, Chinese Academy of Sciences, Beijing, China; 5 Department of Psychology, University of Chinese Academy of Sciences, Beijing, China

**Keywords:** Molecular neuroscience, Psychiatric disorders

## Abstract

Depression may be associated with reduced monoamine neurotransmission, particularly serotonin and norepinephrine (NE). Reuptake of NE by the norepinephrine transporter (NET) is the primary mechanism by which many of the antidepressants are high-affinity substrates for NET. This study aimed to examine the effect of lentivirus-mediated shRNA targeting NET in locus coeruleus (LC) on depression-like behaviors of rats. We randomly assigned 60 male Wistar rats to 6 experimental groups: (1) Control group: without chronic unpredictable mild stress (CUMS) and without NET-shRNA treatment; (2) shRNA group: without CUMS + NET-shRNA; (3) CUMS group: 3-week CUMS without NET-shRNA; (4) CUMS + nonsense shRNA group; (5) CUMS + amygdala (Amy)-shRNA group; (6) CUMS+ locus coeruleus (LC)-shRNA group. First, recombinant lentiviral vector expressing shRNA (ShRNA-629, ShRNA-330, ShRNA-1222, ShRNA-1146 or ShRNA- negative control) against NET were produced, and their efficiency in knocking down of NET in PC12 cells were assessed by Q-PCR and western blot analysis. Second, shRNA was injected into the rat LC bilaterally to investigate whether it could prevent the depressive-like behavior induced by 3-week CUMS. Third, we tested the depressive-like behavior of the rats in the forced swimming test, the open field test, the sucrose preference test, as well as the body weight gain at the end of the seventh week. Finally, the protein expressions of NET was measured by western blot and the NE levels were measured by high performance liquid chromatography. Q-PCR and western blot showed that the ShRNA-1146 had the best interference efficiency targeting on NET in PC12 cells (*p* < 0.01). Compared to the depression model group, the immobility time in the forced swimming test was significantly reduced (*p* < 0.01), but the sucrose preference and the total scores in the open field test were significantly increased (all *p* < 0.01) in the group treated with shRNA in LC. Furthermore, compared with the depression model group, NET levels were significantly decreased (*p* < 0.01), but NE levels were significantly increased in the group treated with shRNA in LC (*p* < 0.05). Our findings suggest that Lentivirus-mediated shRNA targeting NET in LC downregulated NET both in vitro and in vivo, resulting in a significant decrease in depressive-like behavior of rats.

## Introduction

Major depressive disorder (MDD) is a common, serious, life-threatening mental illness, whose incidence is high, affecting 120 million people around the world^1^. After antidepressant treatment, 20–35% patients still have residual symptoms, and their social functions or occupational abilities are impaired. For patients with 3 episodes and without maintenance treatment, the risk of future relapse was almost 100%^2^. Although the underlying pathophysiological mechanisms of depression have not been clarified, animal and human studies have shown that intracerebral neurotransmission dysfunction such as serotonin (5-HT), norepinephrine (NE) and dopamine (DA) plays a pivotal role in the development of depression^3^. Many preclinical and clinical studies have shown that NE involves extensive physiological activities such as learning, memory, awakening and sleep, all of which are important for mental illness^4^. These activities are particularly important for depression and stress-induced autonomic regulation^5,6^. Normal noradrenergic system function may cope with and adapt to stress appropriately. There is sufficient evidence that failure to properly regulate stress response is a key factor in the pathophysiological mechanisms of depression^7,8^. Some studies have shown that NE is overreacting in MDD patients^9–11^. Noradrenergic dysfunction may cause some depressive symptoms in MDD patients, such as lowered noradrenergic function may result in reduced attention, lack of energy, fatigue and mental and psychological slowdowns^12,13^. The central noradrenergic pathways originate from the locus coeruleus (LC), and is primarily projected to the frontal cortex, as well as to the limbic system, including its main components such as the amygdala, hippocampus and hypothalamus, all of which are associated with mood and cognition^14^. These NE brain targets and associated functions are also modified in depressed patients, which include appetite, pleasure, sexual satisfaction, aggression and response to pain^15,16^.

A recent positron emission tomography (PET) study showed that depressed patients who were treated with nortriptyline, a NET-selective tricyclic antidepressant (TCAs) required more than 50% occupancy of central NET for antidepressant efficacy^17^. Since NET turnover rates are decreased in the blood lymphocytes of MDD patients than of normal individuals^18^, altered NET expression also may occur in the brain and are implicated in the pathophysiology of major depression through reduced NE and 5-HT levels in the synaptic cleft^19^. Moreover, selective serotonin reuptake inhibitors (SSRIs) and norepinephrine reuptake inhibitors can inhibit 5-HT and NE transport and elevates their levels in the synaptic cleft, which are commonly used in the treatment of depressive symptoms^20,21^. Antidepressants, such as reboxetine, desipramine, amitriptyline and nortriptyline, are high-affinity substrates for NET, and with repeated administrations these medications downregulate the level of NET^22,23^. The combined serotonin and norepinephrine reuptake inhibitors (SNRIs) including venlafaxine, milnacipran, and duloxetine also exhibit antidepressant effects at least comparable to those of SSRIs in achieving remission^24^. However, these drugs need to be used for a long-term period before clinical improvement occurs, and only 1/3 of these patients will completely relieve depressive symptoms, resulting in partial or incomplete clinical responses in most people^25,26^. Therefore, how to improve the efficacy and rapid effects of antidepressant treatment becomes urgent.

Gene therapy is a treatment method in which nucleic acids are delivered to the cells of the patients as a drug for treating disease^27^. This gene therapy also has unique advantages in specifically targeting pathological brain regions and pathogenic genes^28^. The use of viral vectors to transfer specific genes for target sites in humans is a promising approach. Lentiviral vectors can allow stable and long-term expression of transgenes in non-mitotic cells, which have been widely utilized in neurological diseases^29^. For example, SiRNA treatment for Parkinson’s disease^30^, Huntington’s disease^31^ and Alzheimer’s disease^32^ are in the preclinical stage of research. Lentiviral vectors have already undergone relevant clinical trials in HIV, thalassemia, X-linked adrenoleukodystrophy (ALD), and early-onset metachromatic leukodystrophy (MLD), without serious adverse events^33^. For example, Alexander et al. reported that restoration of p11 expression specifically in the nucleus accumbens of p11 knockout mice normalized depression-like behaviors^34^, suggesting the importance of p11 protein in the depression by application gene therapy. Therefore, a potential gene therapy targeting the NET gene might have therapeutic value for depressed patients as an alternative or augmentation for long-term or even lifelong medications.

In this current study, we used the targeted delivery of a recombinant lentiviral vector expressing shRNA that was directed against NET (NET-shRNA) to downregulate NET expression selectively in the LC. The main purpose was to investigate whether NET-shRNA silenced NET expression/function might evoke fast and robust antidepressant-like responses in the model of depression induced by chronic unpredicted mild stress (CUMS) in rats. We hypothesized that NET-shRNA would produce significant antidepressant-like responses in the rat model of depression.

## Materials and methods

### Animals

We purchased 60 three-month-old male Wistar rats from SLAC Company (Shanghai, China). We placed the rats in plastic cages at 20–22 °C ambient temperature and 50-60% moisture, and allowed them free access to food and water, while keeping them in a 12-h light/12-h dark cycle. Before the behavior test, the rats were adapted to the environment for 30 min. We obtained approval for all animal procedures involved in this study from the Committee of Animal Protection University of Suzhou University, and carried out all experiments in accordance with the guidelines developed by the National Institutes of Health Animal Use and Care.

Rats were randomly assigned into 6 groups, with 10 rats per group: (1) Control group: without chronic unpredictable mild stress (CUMS) and without NET-shRNA treatment; (2) shRNA group: rats were injected with NET-shRNA into the LC bilaterally without CUMS; (3) CUMS group: rats were treated with CUMS for 3 weeks without shRNA treatment; (4) CUMS + nonsense shRNA group: rats were injected with nonsense shRNA into the LC bilaterally after 3 weeks of CUMS; (5) CUMS + Amy-shRNA group: rats which were injected with NET-shRNA into the amygdala (Amy) bilaterally after 3 weeks of CUMS; (6) CUMS + LC-shRNA group, rats were injected with NET-shRNA into the LC bilaterally after 3 weeks of CUMS. After the behavior tests, all the animals were sacrificed under deep anesthesia, and the brains were collected for further biochemical analyses. After the behavioral tests were completed, all animals were given deep anesthesia, and then were killed, with their brains being collected for further biomarker analysis.

### Viral vectors

Since there is no published shRNA sequence that can effectively silence rat NET, we designed and screened the shRNA that can effectively silence the gene. First, the full length of mRNA sequence of SLC6A2 gene of rat was found in NCBI Genebank, and then according to the basic principle of designing shRNA as a template^35^, we obtained 4 target sequences of ShRNA-629, 330, 1222 and 1146, which were designed using the Ambion application (Ambion, Austin, TX, USA). Meanwhile, lentivirus expressing shRNA against NET, which were constructed of transfer vector [LV3 (H1/GFP/PURO)] and three helper vectors (pGag/Pol, pRev and pVSV-G), together with nonsense shRNA as negative control (shRNA-NC) were purchased from GenePharma (GenePharma, Shanghai, China)

The shRNA sequences were depicted as below:

ShRNA-629: 5′-GGCGACCATACCAAATACTCC-3′;

ShRNA-330: 5′-CATATACACTGTTCCTCATCA-3′;

ShRNA-1222: 5′-CATTTCTACTCTGTCGGGATC-3′;

ShRNA-1146: 5′-CCCATGAACATAAAGTCAAG-3′;

ShRNA-NC: 5′-TTCTCCGAACGTGTCACGTTTC-3′.

### Cell culture and transfection

PC12 cells were cultured in DMEM (Gibco, USA) containing 10% fetal bovine serum (Gibco, USA), which was maintained at 37 °C in 5% CO_2_. Vector constructs (10 μl at 1 × 10^8^ TU of titer per milliliter) were transfected into the cells using Polybrene reagent when cells were 90% confluent. After culturing for 72 h, their efficiency in knocking down of NET in PC12 cells was assessed by Q-PCR and western blot analysis.

### Western blot

The protein extract was isolated on 10% SDS-polyacrylamide gel and transferred to PVDF membrane. The primary antibody was a rabbit polyclonal antibody against NET (278990A4.4-P, Alpha Diagnostic, USA; 1:300), mouse monoclonal antibody against actin (YT0096, ImmunoWay Biotechnology, USA; 1:1000). The second antibody was a goat anti-rabbit IgG (111-126-144, Jackson ImmunoResesrch, USA; 1:5000), and a goat anti-mouse IgG (115-155-003, Jackson ImmunoResesrch, USA; 1:5000). Western blotting was quantified using ImageJ software to analyze the intensity of grayscale images. The images were cropped for demonstration.

### Q-PCR

We used a 7500 Real-Time PCR machine (Applied Biosystems, Foster city, CA, USA) to carry out real-time PCR, which contained 1 μl primers (10 μM), 50 ng cDNA and 10 μl of 2XSYBR Green PCR Master Mix (Roche) with a final 20 μl volume. Using GAPDH as internal controls, we standardized the data to GAPDH and calibrated the data with control cDNA. We calculated the relative expression ratio by using the 2^−ΔΔCt^ method. The sequences of primers were depicted as follows: NET Primer F: 5′-GAGTGGCCTACGGAATCACC-3′; NET Primer R: 5′-TCAATGGTGCTGGACCTGAC-3′; GAPDH Primer F: 5′-GGGAAACTGTGGCGTGAT-3′; GAPDH Primer R: 5′-GGGTGTCGCTGTTGA-3′.

### Chronic unpredictable mild stress procedure (CUMS)

We conducted typical CUMS regiment, which was described in details in the previous report^36^. Briefly, the CUMS group was exposed to various unpredictable stressors and was randomly given one kind of stimulations every day, including (1) fasting (24 h), (2) water fasting (24 h), (3) all night lighting (24 h), (4) strange crowded environment (24 h), (5) wet cushion (18 h), (6) squirrel cage tilt (45 °, 24 h), (7) noise stimulation (60 Hz,1 h), (8) cold water swimming (12 °C, 5 min), (9) hot water bath (45 °C, 5 min), (10) clip tail (1 min). The same type of stimulation did not appear continuously, so that the rats could not predict the occurrence of the stimulation. The CUMS lasted 21 days and was stimulated continuously after injection of lentivirus.

The control rats were kept in a separate room, without contact with the stressed rats. They remained undisturbed except for the necessary routine procedures, such as cage cleaning.

### In vivo stereotaxic microinjection

The shRNA was injected bilaterally into the rat as previously described^37^ with minor modifications. First, the animals were anesthetized with 10% chloral hydrate at a dose of 400 mg/kg bodyweight, with the bregma and lambda exposed. Two burr holes (1 mm in diameter) were drilled into the skull to the dural level using a stereotactic device (David Kopf Instruments, Tujunga, CA, USA) according to the coordinates of the LC. The Hamilton syringe loaded with lentiviral vector was slowly lowered to the LC region (AP = −9.8 mm, LAT = ± 1.15 mm, and *V* = −6.0 mm). Once the desired depth was reached, 2 μl of recombinant lentivirus (1 × 10^8^ TU/ml) was delivered to the LC at a rate of 2 μl/10 min^37–39^. For maximum diffusion purposes, the syringe needle was placed at the injection site for an additional 5 min. Moreover, the incision was sutured and sterilized, and the rats were placed on an electric heating pad to maintain their body temperature before recovery. The CUMS + nonsense shRNA group was microinjected with a lentiviral cassette carrying only eGFP. However, the syringe of the CUMS + Amy-shRNA group were lowered bilaterally to the amygdala region (AP = −2.8 mm, LAT = ± 4.8 mm, and *V* = −8.5 mm). The delivery of the lentiviral vector to the LC was verified, and microinjection of the LC region was confirmed by detecting the fluorescence of eGFP under a fluorescence microscope.

### Body weight

We measured the body weights of the rats weekly throughout the experiment.

### Behavioral evaluation

**Fig. 1:**
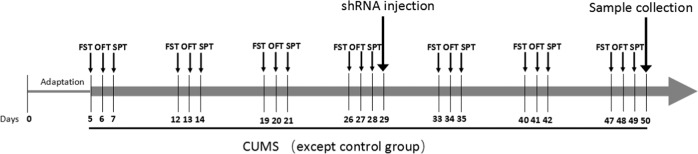
Experimental flow chart shows when behavioral tests were performed. The behavioral tests included forced swimming test (FST), open field test (OFT) and sucrose preference test (SPT). *CUMS* chronic unpredictablemild stress procedure.

All behavioral tests were conducted in order every week and were performed by the same rater, which was presented in Fig. 1.

### Sucrose preference test (SPT)

The SPT was conducted as reported previously^40^. We trained rats to consume sucrose solution prior to the CUMS procedure. During the training period, the first 72 h of sucrose solution was exposed to no water or food. For baseline assessment, the consumption of sucrose solution intake was performed 3 times within 7 days. Food and water deprivation was carried out for 12 h prior to the sucrose preference test. After deprivation, the animal could choose to drink from a 1% sucrose solution or a tap water bottle. In order to avoid potential side-preference effects, the position of the two bottles was switched every 6 h. After 24 h, the consumption of the sucrose solution, tap water and total liquid intake was estimated by weighing the bottles. The preference for sucrose was measured as the ratio of the consumed sucrose solution to total liquid intake. The sucrose preference was determined using the formula: sucrose preference = sucrose intake / (sucrose intake + water intake) × 100%. The test was monitored weekly during the experiment.

### Forced swimming test (FST)

After 3 weeks of CUMS exposure, FST was performed as previously reported^41^. In brief, animals were individually placed in a cylindrical container (40 cm in diameter × 80 cm in height) at a water depth of 45 cm (24 ± 1 °C) and then forced to swim for 6 min. The immobility time for the final 4 min was recorded. The total immobility time was measured according to the time it took for the rat to float without struggling, and only minor movements were performed to keep the head above the water surface. Technical observers were unaware of animal groups.

### Open field test (OFT)

The OFT was conducted as reported previously^42^. The open field is made up of a square arena (80 × 80 cm^2^) separated by a white floor into 25 squares (16 × 16 cm^2^), surrounded by opaque walls which are 40 cm high. In a dimly lit room, both line crossings (place four claws into a new square) and rearing (lift two front claws from the floor) were recorded for more than 5 min. To remove odor clues, the apparatus was cleaned with 5% ethanol after each test.

### Tissue harvesting and analysis

After all behavioral tests were completed, the rats were intoxicated with isoflurane and sacrificed by decapitation. The frontal cortex, hippocampus, amygdala and LC were isolated as previously described^43,44^. We dissected the brain region on the ice. Protein expression of NET was measured by western blot. The detailed procedure for western blotting was performed as previously described^44^.

### High performance liquid chromatography (HPLC)

Norepinephrine levels in the brain region were measured according to previous studies^40^. Add 200 ml of 0.4 M perchloric acid before the homogenization of the issues. The homogenate was centrifuged at 10,000 xg for 15 min. Perchloric acid (0.4 M) was added up to 1 ml and then injected into the HPLC system. Four rats in each group were used to analyze norepinephrine levels.

### Statistical analysis

All data were represented as mean ± SEM. Group differences were examined by one-way analysis of variance (ANOVA), followed by the Fishers Least Significant Difference (LSD) test. We used two-tailed significance and p value <0.05 was considered significant. GraphPad Prism 5.0 and SPSS 20.0 were used for statistical analysis.

## Results

### Efficacy of NET downregulation procedure

**Fig. 2:**
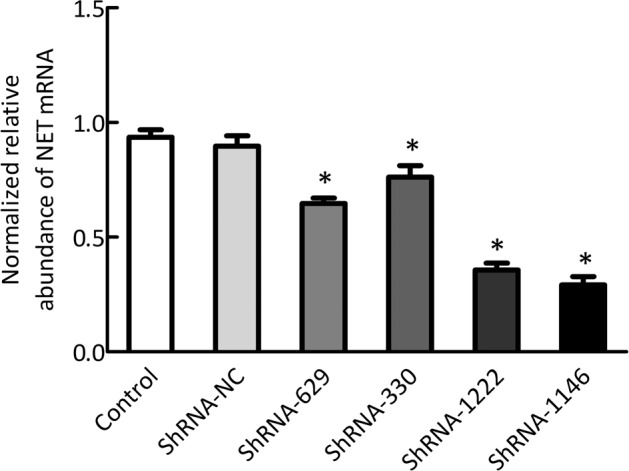
Q-PCR showed the relative expression of NET mRNA in PC12 cells after NET-shRNA interference (*n* = 3). *p < 0.01 vs control group.

We produced recombinant lentiviral vector expressing shRNA against NET (NET-shRNA) and nonsense shRNA as a negative control (ShRNA-NC). We assessed the efficiency of the NET-shRNA in knocking down of NET in PC12 cells using Q-PCR and western Blot. Compared to the control group, the relative expressions of NET mRNA in PC12 cells after NET-shRNA interference were 64.72 ± 4.01, 76.15 ± 8.64, 35.68 ± 5.27, 29.18 ± 6.22% on the ShRNA-629, ShRNA-330, ShRNA-1222, and ShRNA-1146 groups, respectively (all *p* < 0.05; Fig. 2). We found that there was no significant difference in NET mRNA levels between the control group (93.60 ± 5.65%) and the ShRNA-NC (89.73 ± 7.65%) group (*p* > 0.05).

**Fig. 3:**
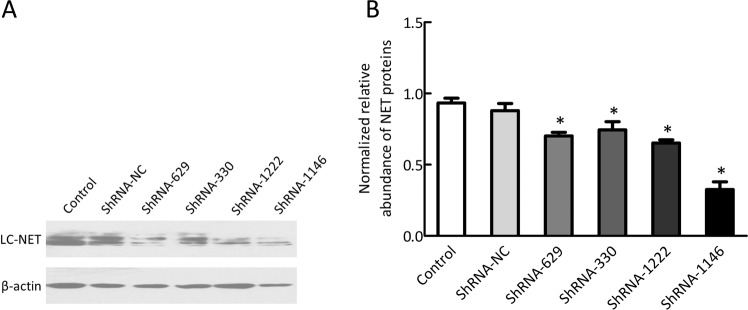
Western blot showed the relative expression of NET protein in pC12 cells after NET-shRNA interference. **a** Representative western blot of NET levels in each group; **b** Quantitative analysis of western blot shown in **b** (*n* = 3). *p < 0.01 vs control group.

Compared to the control group, the relative expressions of NET protein in PC12 cells after NET-shRNA interference were 70.22 ± 4.24, 74.49 ± 9.97, 65.14 ± 3.93, and 32.59 ± 9.17% on the ShRNA-629, ShRNA-330, ShRNA-1222 and ShRNA-1146 groups, respectively (all *p* < 0.05; Fig. 3). No significant difference in NET protein levels was noted between the control group (93.31 ± 5.94%) and ShRNA-NC group (87.90% ± 8.73%) (*p* < 0.05). It appeared that the ShRNA-1146 had the best interference efficiency targeting on NET, which was then selected to be injected into the rat LC bilaterally to investigate whether it might prevent the CUMS-induced depressive-like behavior in the following experiments.

### Effects of microinjection NET-shRNA into LC on depression-like behavior and the body weight

**Fig. 4:**
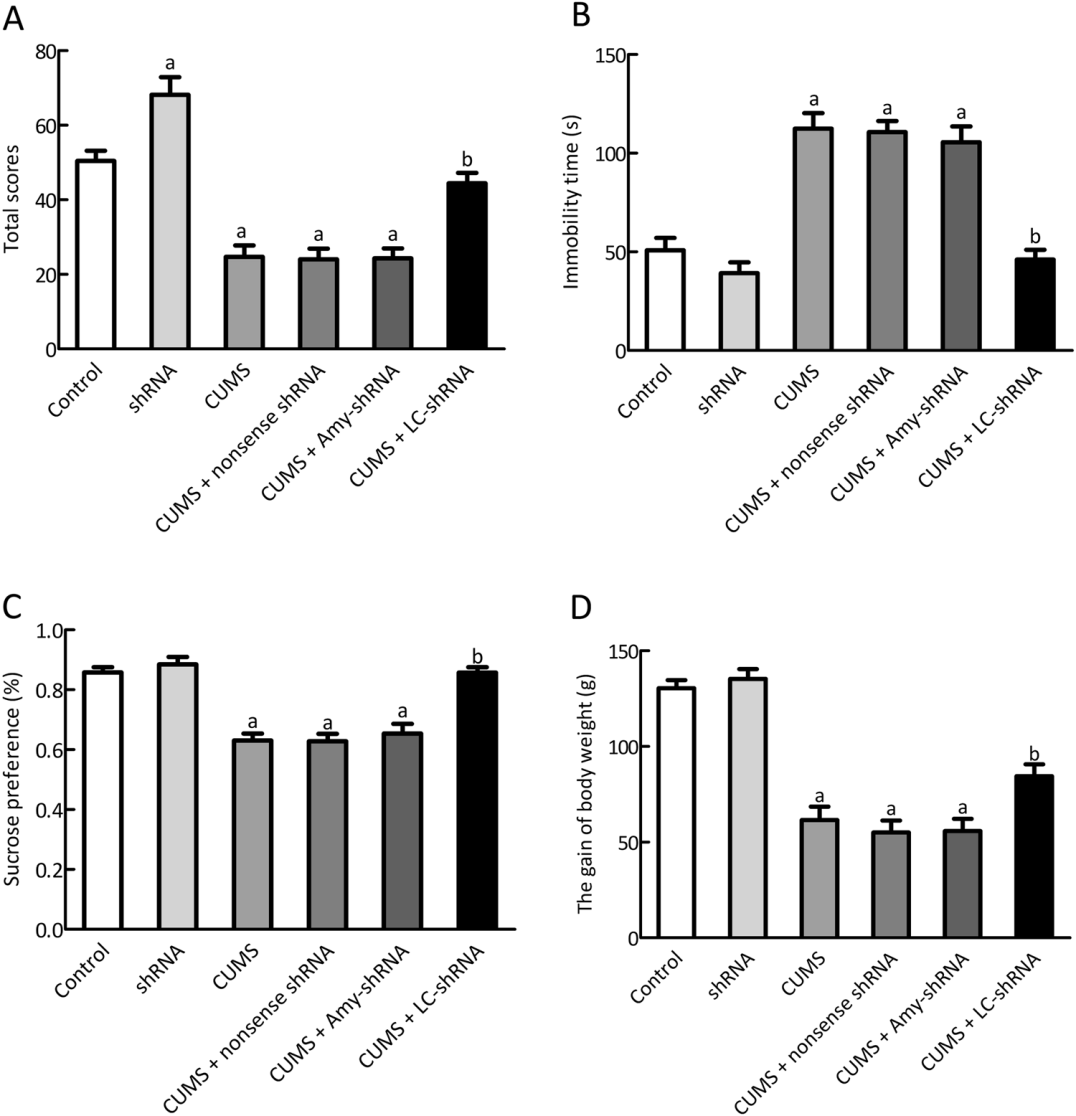
Effects of microinjection NET-shRNA into LC on depression-like behavior and the gain of body weight. **a** In the open field test, the total scores was recorded; **b** In the forced swimming test, the immobility time was recorded; **c** In the sucrose preference test, the sucrose preference was recorded; **d** The gain of body weight in 3 weeks after the shRNA injection. *n* = 10; ^a^*p* < 0.01 vs control group, ^b^*p* < 0.01 vs CUMS group.

We conducted all behavioral tests weekly after the CUMS. Consistent with previous results, the peak reduction in depressive-like behaviors in the rats occurred at 3 weeks after microinjection of net-shRNA into LC^45–47^. The open field test, which assessed spontaneous locomotor activity in animals using a previously reported experimental method^42^, showed statistically lower scores in the CUMS group compared with the control group during the seventh week (*p* < 0.01). However, the CUMS + LC-shRNA group showed significantly greater open field scores compared with the CUMS group (*p* < 0.01; Fig. 4a). Furthermore, the ShRNA group showed significantly greater open field scores than the control group (*p* < 0.05; Fig. 4a). The CUMS and ShRNA-NC groups showed no significant difference in their open field scores (på 0.05; Fig. 4a).

In the forced swimming test, the immobility time at the end of the seventh week was significantly greater in the CUMS than the control group (*p* < 0.01; Fig. 4b). However, the immobility time was significantly less in the CUMS + LC-shRNA than in the CUMS group (*p* < 0.01; Fig. 4b). The CUMS and ShRNA-NC groups did no show significant difference in their immobility times (*p* > 0.05; Fig. 4b).

During the seventh week the CUMS group showed significantly lower sucrose preference than the control group (*p* < 0.01; Fig. 4c). However, the CUMS + LC-shRNA group showed significantly greater sucrose preference than the CUMS group (*p* < 0.01; Fig. 4c). The CUMS and ShRNA-NC groups did not display significant difference in sucrose preference (*p* > 0.05; Fig. 4c).

We measured weight change every week, which may be an alternative indicator of appetite changes and a diagnostic criterion for depression^48,49^. At the week 7, as shown in Fig. 4d, the CUMS group had significantly fewer gain of bodyweight than the control group (*p* < 0.01). However, the gain of body weight was statistically greater in the CUMS + LC-shRNA group compared with the CUMS group (*p* < 0.05). The gain of body weight showed no significant difference among the CUMS, ShRNA-NC and CUMS + Amy-shRNA groups (all *p* > 0.05).

### Effects of the NET-shRNA microinjection into LC on NET protein

**Fig. 5:**
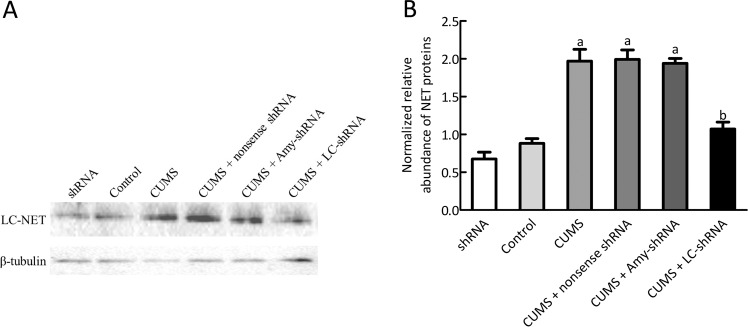
Western blot showed the relative expression of NET protein in LC after NET-shRNA microinjection. **a** Representative western blot of NET levels in each group; **b** Quantitative analysis of western blot shown in **b** (*n* = 3). ^a^*p* < 0.01 vs control group, ^b^*p* < 0.01 *vs* CUMS group.

Figure 5 shows western blot results that demonstrate the relative expression of NET protein in LC after NET-shRNA microinjection. NET expression was statistically greater in the CUMS compared with the control group (*p* < 0.01). NET expression was statistically less in the CUMS + LC-shRNA than the CUMS group (*p* < 0.01). No significant difference in NET expression was observed between the control and ShRNA groups (*p* > 0.05) or among CUMS group, CUMS + nonsense shRNA group and CUMS + Amy-shRNA group (all *p* > 0.05).

### Effects of the NET-shRNA microinjection into LC on NE levels

**Fig. 6:**
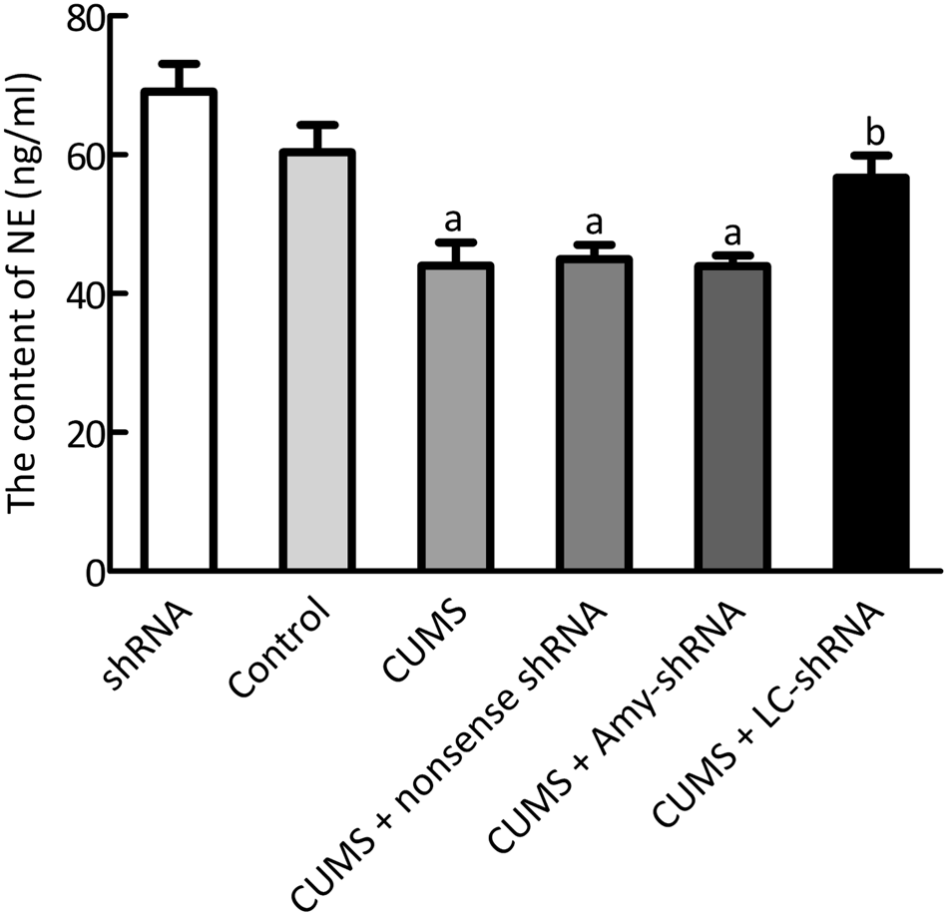
HPLC showed NE levels in LC after NET-shRNA microinjection (*n* = 4). ^a^*p* < 0.05 *vs* control group, ^b^*p* < 0.05 *vs* CUMS group.

Figure 6 shows the HPLC results for NE levels in LC after NET-shRNA microinjection. There were significantly lower NE levels in the CUMS compared with the control group (*p* < 0.01), while there were significantly greater NE levels in the CUMS + LC-shRNA compared with the CUMS groups (*p* < 0.01). No significant difference in NE levels were noted between the Con and ShRNA groups, or among the CUMS, the CUMS + nonsense shRNA and the CUMS + Amy-shRNA groups (all *p* > 0.05).

## Discussion

This study successfully used targeted delivery of a recombinant lentiviral vector expressing shRNA directed against NET (NET-shRNA) to downregulate NET expression selectively in the LC. This decreased NET expression occurred quickly after delivery of the NET-shRNA, and it had robust functional consequences in several rat models of antidepressant-like responses. Using this CUMS model of chronic stress clearly led to dysregulation of NE nervous system, which may be an important rodent model for depression and other human disorders such as post-traumatic stress disorder, which also manifests a significant dysregulation in NE and can respond to antidepressant medications. Thus, this rat model using a partial knockdown of the NET gene can be helpful as a pathophysiological model of human psychiatric diseases.

These rodent model behavioral responses to partial knockdown of the NET gene are consistent with clinical MDD treatments using high-affinity blockers of NET, such as SNRIs, which enhance the synaptic activity of NE^22^. Because many MDD patients need to continue these medications for their lifetime, this alternative way to maintain NET inhibition through lentiviral vectors rather than lifetime medication adherence holds great promise. A recent study showed that 15 Parkinson’s patients were treated with lentivirus vector-mediated ProSavin by bilateral injection of lentivirus vector into the striatum. Compared with the baseline, the average score of UPDRS- III in all patients increased significantly at 6 and 12 months, and no serious adverse events related to lentivirus or surgery occurred^50^, suggesting that the downregulation of genes by shRNA may last for a long period of time. Overall, this technique can carry multiple exogenous genes into neurons, and those genes can be expressed for long periods of time without causing immune responses^51^. Furthermore, this therapeutic strategy may overcome the slow clinical action and low efficacy of current antidepressants.

### Increased expression of NET induced by chronic stress as component of depression

NET is one of the key candidate genes for single nucleotide polymorphism (SNP) research in the etiology of human depression. Many clinical studies have examined the reduction of NET expression caused by genetic polymorphisms, which have been found to produce effects on the incidence^52^ and severity^53^ of depression, as well as on the efficacy of antidepressants^54^. However, studies are not consistent in these associations^55,56^.

CUMS is a widely used animal model of depression. Compared with the single stressor model, it simulates the stressors of a series of negative life events that patients with depression encounter in their daily lives^57^. In the FST, rats were first exposed to water, and they initially exhibited active escape-related behaviors, such as swimming and climbing, and then passive immobile behavior, which is thought to model behavioral despair and helplessness in human depression^58^. Sucrose preference in the SPT decreases after CUMS and is thought to reflect human anhedonia. A wide range of antidepressant treatments also prevent or reverse this loss of sucrose preference^59^. Rats in the OFT show reduced movements of crossing and forelimb rearing in new environments, which models psychomotor retardation in human depression^42^. The CUMS rodents also have a slow weight gain, which is used as a model of appetite and weight loss in human depression, although the effects of fasting and water deprivation during the experiments confound this interpretation and parallel to human depression. Overall, the CUMS rodent model of chronic stress did produce a range of behaviors that have been considered analogues of depression in humans and support a critical role for NET reduction in the etiology of these behaviors and potentially in human depression.

### ShRNA targeting NET in LC attenuates depression-like behavior

Abnormal function of NE system plays an important role in the pathogenesis and treatment of depression. Locus coeruleus (LC) is the main nucleus of NE neurons. NET reuptake 92% of the NE released from sympathetic nerve into presynaptic membrane by high-affinity active transporter, which plays an important role in fine regulation of NE concentration in synaptic space^52–54^. Previous studies showed that NE alteration in LC may be involved in the psychopathological mechanisms of depression as well as in the treatment of depression^4–6^. In this study, the increase of NET caused by chronic stress may lead to the decrease of NE bioavailability, which may be involved in the pathogenesis of depression. Therefore, shRNA targeting downregulation of NET, in LC can increase the concentration of NE and improve the depression-like behavior of rats.

In this study, a single 2 μl NET-shRNA injection into the rat LC significantly downregulated NET and increased NE levels for at least 4 weeks in this specific brain region without affecting other regions such as the amygdala. This gene silencing effect reversed many changes induced through the CUMS chronic stressors as shown by an increased rate of body weight gain, increased interest, increased autonomous behavior, and reduced hopeless behavior. These rodent models of human depression-like behaviors further suggested the important role of NET in the pathophysiological mechanism of human depression. In addition, the NET-shRNA had no significant effect on normal rats, which maintained NE system homeostasis. The number of CUMS-induced changes that were reversed was notable considering that we only disrupted the NE system and only in one brain region, and additional antidepressant mechanisms are likely involved in clinical responses, such as enhanced NE neurotransmission in other brain regions that are involved in MDD.

### Viral vector-mediated knockdown method versus traditional pharmacological and genetic procedures

Our findings in this study have demonstrated the important implication of NET expression in the LC in the regulation of mood states. Our results also showed that viral vector-mediated knockdown methods have particular advantages over traditional pharmacological and genetic approaches commonly utilized to change the NE system. Importantly, the NET gene in humans and rats is highly homologous^60^. Previous studies showed that NET knockout mice (NET KO) were similar to their wild-type littermates, when treated with antidepressants in behavioral^61^ and neurophysiological assessments^62^, and a variety of antidepressants had no effect on NET KO mice^63^. However, since complete absence of NET does not occur in viable human embryos, behavioral and pharmacological responses in NET KO mice may be hard to explain because compensatory developmental changes may emerge due to defective genes^63^.

The NET-shRNAs we constructed can achieve stable silencing of NET genes *in vitro*. The gene silencing efficiency of ShRNA-1146 is the highest among the four shRNA sequences, and it was selected to establish a partial knockdown NET gene rat model, which mimics the pathophysiological mechanism of human diseases. This similarity is based on four factors. First, the localized knockdown in the LC avoids the potential compensation for genetic defects or abnormal neuronal development, which occurs in the development of traditional NET KO mice. Second, the use of traditional constitutive KO models lacks neuroanatomic specificity, while this NET-shRNA rat model allows targeting of more precise brain regions. The NET-shRNA rat lesion is more precise in time and space. Third, when antidepressants are administered acutely into precise brain regions, those doses are usually much greater than the associated physiological brain levels in human body, and acute dosing does not provide a chronic increase in NE release induced by human antidepressant treatment. Fourth, although the specificity of antidepressants such as reboxetine is high in localized brain applications, specificity is not absolute and the dopamine transporter in the same neuroanatomical location also can be inhibited. Further, it is worth mentioning that CUMS + Amy-shRNA group was set up to demonstrate the injection into the locus coeruleus rather than other brain regions. The noradrenergic neurons in locus coeruleus account for more than half of the total amount in the brain^64^. Although a small number of norepinephrine nerve fiber bundles project to the basolateral nucleus of the amygdala, the NE in the amygdala accounts for a very small proportion of the total NE^65^. Thus, chronic stress may mainly affect the locus coeruleus and prefrontal cortex NE. Therefore, after NET-shRNA injection into bilateral amygdala, there was no change in NET expression and NE level. At the same time, stress may increase the NE of the basolateral nucleus of the amygdala, and its compensatory effect may lead to no significant changes in NET expression and NE levels. However, these are just our speculations and need to be verified in new studies. In addition, in this study, there was no significant change in body weight and behaviors in Con+shRNA group, suggesting that NET-shRNA had no significant effects on weight and behaviors of the normal rats, which may be due to the endogenous homeostasis regulation of NE system balance in the brain of normal rats. However, the underlying mechanism needs to be further explored.

The present data support that the enhanced NET activity is mediated via NET, suggesting that NET is responsible for the observed NE transport in the NET-knockdown rats. However, besides norepinephrine, NET could also transport dopamine (DA). Moreover, considering the close relationship between DA and NE systems, the possible effects of NET-shRNA on DA pathway will need to be clarified. Because of the lack of DAT in the prefrontal cortex, DA is inactivated by reuptake of NE by NET. At present, selective norepinephrine reuptake inhibitors (NRI) such as reboxetine and atomoxetine have been widely used. Reboxetine can increase the NE in the prefrontal cortex through the inhibition of the reuptake of NE, and also increase the transmission of DA in the prefrontal cortex^66^. However, the reuptake effect of reboxetine on NE was about 100-fold and 1000-fold higher than that on 5-HT and DA, respectively, and had almost no clinical significance on the reuptake of 5-HT and DA^67^, whereas studies support that DAT is not a likely compensatory mechanism for NE uptake^68^. Neither 5-HTT nor DAT provided a mechanism for enhanced NE transport in the NET + / − mice^69^. Therefore, the shRNA targeting effect on NET gene in this study may have a smaller effect on 5-HT and DA reuptake.

Dopaminergic neurons were mainly located in substantial nigra pars compacta, ventral tegmental area and arcuate nucleus. However, in this study, NET-shRNA was stereotaxically injected into locus coeruleus, so it may have little effect on DA system. At the same time, it can also avoid the side effects of NRI such as reboxetine on the whole brain and even peripheral norepinephrine system and dopaminergic system as constipation, urinary retention and other side effects^70^. In future experiments, DA will be detected to confirm the effect of NET-shRNA on DA.

It is worthy of mentioning that the concept of treating depression with the NET gene therapy seems interesting, but still distant, not only technical problems that need to be overcome, but also neurobiological consequences, such as compensation within other transporters, which may entail other functional disruptions occurring in the body. Thus, silencing NET in the LC may affect the level of protein of this transporter in other regions of the brain, which may still entail changes in the quantity/functions of the other transporters. Therefore, it should be taken into account and checked in the future.

Limitations of the current study involve neuroanatomic specificity, the extent of NET activity reduction and the longer term adjustments that might occur from this NET inhibition. We could not exclude the possibility that the viral vector could have affected more than one brain regions of LC, or that the precise extent of NET silencing was different between individual rats or among the groups. We will examine this possibility in future studies by using new non-invasive imaging techniques to track the expression of the NET gene within an individual rat and the proliferation of lentiviral vectors beyond the site of injection. Moreover, determination of duration of single NET-shRNA injection and its long-term effect is important. Unfortunately, we did not carry out the experiments for long-term effect of NET-shRNA injection, which should be remedied in future study. Thus, future studies will explore less invasive treatments and more appropriate therapeutic doses, as well as the issue of duration of these reductions in NET activity, since homeostatic mechanisms in the brain might upregulate the production of NET, as well as the NET-shRNA effect attenuating as the NET-shRNA is degraded over time. In addition, NE also plays an important role in anxiety. In this current study, we did not measure the anxiety-like behavior and did not distinguish the behavior of rats in the experiment was depression-like behavior rather anxiety-like behavior, which should be remedied in the future studies. Finally, the rats were limited to only males and cannot be applied to females.

In summary, lentivirus-mediated shRNA targeting NET in LC downregulated NET both in vitro and in vivo, which resulted in a significant decrease in depressive-like behaviors of rats, opening a way for further translational studies.
